# Elder Mistreatment and Psychological Well-Being among Older Americans

**DOI:** 10.3390/ijerph17207525

**Published:** 2020-10-16

**Authors:** Ronald W. Berkowsky

**Affiliations:** Health Science Program, California State University Channel Islands, 1 University Dr, Camarillo, CA 93012, USA; ronald.berkowsky@csuci.edu; Tel.: +1-805-437-2043

**Keywords:** elder mistreatment, elder abuse, psychological well-being, eudaimonic well-being, older adults

## Abstract

Elder mistreatment is a major public health issue both in the US and around the globe. While extensive research has elucidated the association between elder mistreatment and health in older adults, little is known about the relationship between elder mistreatment and more eudaimonic measures of psychological well-being. Using data from the 2011 wave of the Wisconsin Longitudinal Study, this project examined the association between older adults’ experience with varied forms of elder mistreatment and eudaimonic dimensions of psychological well-being including autonomy, environmental mastery, personal growth, positive relations with others, purpose in life, and self-acceptance. Ordinary least squares regression analyses found significant associations between experiences of elder mistreatment and psychological well-being. In particular, older adults who reported feeling that someone was too controlling over their daily lives and older adults who reported making donations to organizations they later worried were not legitimate reported significantly lower scores on all six psychological well-being dimensions. The results of this study suggest the negative effects of elder mistreatment can extend to more eudaimonic feelings of well-being, and programs designed to treat victims of elder mistreatment should incorporate strategies that help strengthen psychological well-being.

## 1. Introduction

The purpose of the project described herein was to examine the relationship between elder mistreatment and eudaimonic, rather than hedonistic, dimensions of psychological well-being. Elder mistreatment, or elder abuse, constitutes any action or inaction that jeopardizes the health or well-being of an older adult [1]. Most scholars identify physical abuse, emotional abuse, sexual abuse, neglect, and financial exploitation of older adults as forms of elder mistreatment [2], though no single definition exists that simultaneously defines the dimensions of elder mistreatment while also being applicable across varied professions and contexts (e.g., research, law, critical care) [3]. Approximately 10% of older Americans have experienced at least one form of elder mistreatment [2], and globally approximately 15.7% of all older adults have experienced at least one form of mistreatment translating to approximately 141 million people [4]. Due in part to this heightened prevalence and the likelihood that these may be underestimates due to underreporting [5,6], elder mistreatment has been cited both in the US and around the globe as a major public health concern.

The impacts of elder mistreatment on the physical and mental health of older adults have been well-documented. Elder mistreatment has shown significant associations with increased mortality [7,8,9,10,11], declines in functional health [12], increased risk of disability [11], increased depression [13,14,15,16], increased feelings of anxiety [12,13,14,15], increased incidence of post-traumatic stress disorder [13,14,17], and increased loneliness [12]. Many studies rely on cross-sectional data, but recent work using longitudinal data has elucidated the long-term impacts of elder mistreatment. As an example, analysis of data from multiple waves of the National Social Life and Aging Project showed older adults who experienced verbal or financial mistreatment reported increased anxiety, increased loneliness, and worse functional health five years later compared to those who reported experiencing no mistreatment [12].

Significant correlates and risk factors to elder mistreatment include functional dependence [18,19,20], poor self-perceived health [21,22], cognitive decline [23], and previous exposure to traumatic events [24], among others. It is important to note, however, that correlates may vary depending on the type of mistreatment being examined or the population under investigation. As an example, a study of US Chinese older adults found that high levels of education were associated with elder mistreatment risk [22], but a study of South Korean older adults found low levels of education were predictive of increased risk [17]. Social support has more consistently been found to be a protective factor such that older adults with higher levels of support tend to experience less mistreatment and abuse [19], but even this finding has been disputed by recent longitudinal work [12].

Several theories have been proposed to explain the motivations behind those who mistreat older adults and to explain why older adults may be more susceptible to mistreatment. The most prevalent theories focus specifically on interactions between caregivers and care recipients [25,26]. Caregiver stress theory describes how caregivers of vulnerable older adults may become abusive towards their care recipients as a response to the stresses related to caring responsibilities [26]. Relatedly, social exchange theory posits that in social relationships there is an expectation of an equitable exchange of resources. When applied to elder mistreatment, social exchange theory argues that if an older adult becomes more reliant on their caregiver without a measurable increase in benefit to the caregiver, they may become angry and resentful towards the care recipient, thus increasing the risk of abuse and mistreatment [25]. Social exchange theory also argues that an older adult receiving increased care may feel obligated to provide additional resources (e.g., monetary compensation) to make the caregiving relationship more equitable; this, in turn, may make the older adult more susceptible to exploitation [25,26]. An example of a theory explaining elder mistreatment outside of the caregiving relationship is the political economic theory, which posits that individual independence is reduced as a result of transitioning out of the workforce, making older adults vulnerable to excessive control and abuse [25].

Despite extensive work examining the impacts of elder mistreatment on health, less is known about the association between elder mistreatment and more eudaimonic measures of psychological well-being. Proposed by Ryff in 1989 [27], the eudemonic approach to psychological well-being attempts to address gaps in our theoretical understanding of a fulfilled and happy life [27,28,29,30]. Traditional research on well-being defined the concept as using more hedonistic views of pleasure attainment and the avoidance of pain. This hedonistic approach to well-being, with historical and philosophical roots in Ancient Greece, emphasized happiness, life satisfaction, and positive affect. In contrast, Ryff argued for a more eudaimonic approach (also based on philosophy from Ancient Greece) to well-being, which emphasized self-realization, individuation, development, and function [27,28,31]. In re-evaluating what it means to be well, Ryff distilled six key dimensions of psychological well-being that went beyond feelings of happiness or satisfaction and instead focused on characteristics that contribute to better understandings of the self and that contribute to becoming that better-understood sense of self. These dimensions included autonomy (i.e., the ability to self-determine and live by one’s own convictions), environmental mastery (i.e., the ability to control and manage one’s surroundings and opportunities), personal growth (i.e., feelings of continued development), positive relations with others (i.e., having warm and satisfying connections with others), purpose in life (i.e., having a sense of meaning and direction with one’s life), and self-acceptance (i.e., having a positive attitude towards oneself) [27,28,29,30].

Since Ryff’s identification of these dimensions, numerous studies have examined the relationship between eudaimonic measures of psychological well-being and health among older adults. Particular interest has been paid to purpose in life as a predictor of health, as older adults are at risk for lower levels of this dimension [27,32]. Studies show that among older adults, higher levels of purpose in life were associated with reduced risk of mortality [33,34], reduced risk of cognitive impairment [35], reduced risk of stroke [36], and increased likelihood of engaging in positive and preventative health behaviors [37]. Beyond purpose in life and examining other dimensions, increased psychological well-being has been associated with reductions in the negative impacts of late-life comorbidities [38] and better sleep outcomes among older women [39].

With a demonstrated association between having higher levels of psychological well-being and reporting better physical and mental health, interest has grown in examining ways to promote eudaimonic well-being to positively impact health. As an example, the Lighten UP! eight-week training program was designed to enhance psychological well-being among older adults through facilitated group sessions that involved identifying, reflecting, and savoring positive life experiences [40]. Evaluation of Lighten UP! showed participants enjoyed sustained gains in psychological well-being while also reporting lower depression, anxiety, and hostility [40,41].

To date, little is known about the relationship between elder mistreatment and eudaimonic measures of psychological well-being. One could argue that given the already studied negative impacts of elder mistreatment, eudaimonic measures of psychological well-being would also be negatively impacted. As an example, if someone close to an older adult (e.g., family member, friend, or caregiver) exerts an excessive amount of control over the older adult’s life to the point that they feel limited in what they could do, the older adult would potentially report lower levels of autonomy (i.e., they would feel less independent), environmental mastery (i.e., they would feel less control over the external world), and positive relations with others (i.e., they would feel more isolated and frustrated with interpersonal relationships). To this end, this project sought to determine if a relationship exists between elder mistreatment and psychological well-being. More specifically, this project examined the association between various forms of elder mistreatment and the six dimensions of psychological well-being defined by Ryff to determine if (a) experiences of elder mistreatment correlate with lower psychological well-being and (b) if these correlations vary based on the type of elder mistreatment experienced. Findings may illustrate potentially unmeasured consequences of elder mistreatment which must be accounted for when assisting and treating abuse victims.

## 2. Materials and Methods

Data for this project came from the Wisconsin Longitudinal Study (WLS), a longitudinal investigation of men and women who graduated from a Wisconsin (US) high school in 1957 with principal support from the National Institute on Aging [42,43]. The purpose of the WLS was to investigate and link early life characteristics and experiences (e.g., family background, education, income, experiences with employment, etc.) with later life outcomes (e.g., physical health, mental health, mortality, etc.). The original cohort was based on a 1/3 random sample of all Wisconsin high school graduates born between 1938–1940. Since the initial interview in 1957, graduate respondents have been routinely interviewed (i.e., 1964, 1975, 1993, 2004, and 2011) via mail, telephone, and in-person interviews. Beginning in 1977, the study was expanded to include data from selected siblings (interviewed in 1977, 1994, 2005, and 2011). This project uses publicly available data from the most recent 2011 wave, as this is the only wave where questions were asked of graduates and their selected siblings on experiences with elder mistreatment. The total number of graduates and selected siblings who participated in at least the mail-in portion of the 2011 data collection (from which the outcome and predictor measures were assessed) was 8381.

While the entirety of the graduate sample had entered older adulthood by 2011, some in the sibling sample had not. Given this project’s focus on mistreatment of older adults, the analytic sample was limited to WLS graduate and sibling respondents who were at least 65 years old at the time of the 2011 wave data collection (reducing the analytic sample to 7449). The analytic sample was also limited to those who had valid responses on all predictors and outcomes of interest. These limitations yielded an analytic sample of 6158 older adults, or approximately 83% of the graduate and sibling respondents who had submitted a valid 2011 mail-in survey and who were aged at least 65 years at the time of submission. The primary reason a participant was dropped from the analytic sample was due to missing data on the elder mistreatment predictors—given how elder mistreatment is widely known to be underreported [5,6], this was expected.

### 2.1. Outcomes

The outcomes of this project were eudaimonic measures of psychological well-being as defined by Ryff [27], including autonomy, environmental mastery, personal growth, positive relations with others, purpose in life, and self-acceptance. In the WLS, a series of statements related to these dimensions were presented via a mail-in survey and respondents were asked to indicate (using a 6-point Likert scale) how much they agreed or disagreed with each statement. Five items were used to assess autonomy, environmental mastery, personal growth, and self-acceptance; six items were used to assess positive relations with others and purpose in life. The point values for all items of a given dimension were recorded and summed to produce a final score for that dimension, with higher scores translating to higher psychological well-being.

Missing responses to individual dimension items were imputed as the mean of the valid dimension items prior to summing if respondents had provided responses to at least half of the dimension items. As an example, if a respondent answered three of the five autonomy items, a mean-imputed score based on the three valid responses was calculated for the missing values prior to summing. While mean-imputed scores are not optimal, their use can prevent each summed dimension score from being underestimated due to missing values, and there is evidence that mean-imputed scores can produce reliable results so long as there is sufficient data on the other items of a given dimension [44]. If a respondent had less than half of the items answered in a given dimension, no mean imputation was calculated, and their summed dimension score was considered missing (and thus not used in the analysis). Of the 6158 in the analytic sample, the number of respondents who required mean imputations for an outcome measure was 85 for autonomy, 97 for environmental mastery, 81 for personal growth, 127 for positive relations with others, 125 for purpose in life, and 144 for self-acceptance. Table 1 includes the list of psychological well-being dimensions, the number of items per dimension, a sample item, and the recorded sum ranges.

### 2.2. Predictors

The primary predictors were experiences of elder mistreatment. Eight questions from the WLS 2011 mail-in survey asked respondents to indicate if they had experienced certain instances of mistreatment within the past year. Questions covered varied forms of mistreatment experiences including experiences of excessive control, receiving insults, belongings being seized/withheld, physical abuse, blocked access to everyday needs, and financial exploitation. Responses were coded 0 = *No*, 1 = *Yes*. Mistreatment items and question wording are provided in Table 2.

### 2.3. Demographic Controls

In addition to the primary predictors, demographic variables were included in the analysis as controls. These variables included: age (continuous), sex (0 = *Male*, 1 = *Female*), education (0 = *High school degree or less*, 1 = *At least some college*), marital status (0 = *Not married*, 1 = *Married*), number of household members including the respondent (continuous), employment status (0 = *Not employed*, 1 = *Employed*), and self-rated health (measured on a 5-point scale with 0 = *Very poor health* and 4 = *Excellent health*). The WLS cohort has so few non-White respondents that racial/ethnic data are considered personally identifiable information; as such, this variable was not available in the public data and was not controlled for in the analysis.

### 2.4. Analytic Procedure

Two ordinary least squares (OLS) regression models were run for each outcome to determine the relationship between experiences of elder mistreatment and each individual dimension of psychological well-being (*p* < 0.05). In the first model, each psychological well-being dimension was regressed on the elder mistreatment measures. In the second (full) model, demographic variables were introduced as controls. Analysis was done using SPSS ver. 27 (IBM Corp., Armonk, NY, USA).

## 3. Results

Sample descriptives are presented in Table 3. The mean age was 71.38 years, and the sample was mostly female (53.59%), had attended at least some college after graduating high school (53.75%), and was married (72.22%). The average household size was around two (including the respondent), about a quarter of the sample was employed (27.01%), and the average score on self-rated health (on a scale from zero to four) was 3.00 indicating the sample viewed itself mostly in good health. While 7.49% of the sample reported mistreatment experiences related to excessive control and 14.90% reported receiving insults in the past year, the percentage of respondents who indicated that they experienced the other types of elder mistreatment were relatively low. Regarding the outcome measures, respondents scored an average of 17.75 on autonomy, 19.70 on environmental mastery, 19.50 on personal growth, and 19.51 on self- acceptance (on a scale of 0 to 25). On average, respondents also scored 23.06 on positive relations with others and 21.87 on purpose in life (on a scale of 0 to 30). These scores indicate the sample as a whole enjoyed more positive levels of psychological well-being with particularly strong scores for environmental mastery, personal growth, positive relations with others, and self-acceptance. Autonomy and purpose in life, comparatively, showed the weakest scores.

Prior to the regression analysis, Pearson correlation coefficients were calculated. These coefficients are presented in Table 4 and elucidate relationships that may potentially be observed in the regression analysis. Notably, feeling someone had exerted excessive control over you in the past year and being worried you made a donation to an illegitimate charity both demonstrated significant negative correlations with all six outcome measures. These negative correlations suggest that respondents who reported experiencing these types of elder mistreatment, on average, scored lower on all measures of psychological well-being in the absence of other variables. Education, employment status, and self-rated health also showed significant correlations across all outcomes in the absence of other variables.

### 3.1. Elder Mistreatment Predicting Psychological Well-Being

Table 5 shows the results of the regression models examining the relationship of elder mistreatment and each psychological well-being dimension in the absence of demographic controls. WLS respondents who indicated that they felt there was someone who exerted excessive control over their lives in the past year scored significantly lower on all dimensions of psychological well-being compared to those who reported no experiences of excessive control. Respondents who reported being insulted over the past year scored significantly lower on environmental mastery (−0.715, *p* < 0.001), positive relations with others (−0.723, *p* < *0*.001), and self-acceptance (−0.776, *p* < 0.001). Interestingly, having belongings seized or withheld in the past year was positively associated with autonomy (0.742, *p* < 0.05), such that those who reported these experiences had significantly higher autonomy scores compared to those who did not have their belongings seized or withheld. Having belongings seized or withheld was not significantly associated with any of the other psychological well-being dimensions. Physical abuse was significantly associated with lower scores on environmental mastery (−1.386, *p* < 0.05) and positive relations with others (−2.892, *p* < 0.01) while blocked access to basic needs was not significantly associated with any of the outcomes. Feeling exploited while making a purchase in the past year was significantly associated with lower environmental mastery (−0.572, *p* < 0.05), positive relations with others (−1.057, *p* < 0.01), and self-acceptance (−0.613, *p* < 0.05). Feeling exploited while selling was significantly associated with lower environmental mastery (−1.737, *p* < 0.01), purpose in life (−1.555, *p* < 0.05), and self-acceptance (−1.502, *p* < 0.05). Respondents who felt they may have made a donation to an illegitimate charity in the past year had lower average scores across all psychological well-being dimensions.

The R^2^ values for the models were relatively low, indicating the elder mistreatment items alone did not account for a substantial amount of the variance in psychological well-being scores in the sample. This suggests the inclusion of additional predictors is warranted.

### 3.2. Full Regression Models Introducing Demographic Controls

Table 6 shows the results wherein demographic controls are added to the regression models. Of the control variables, education and self-rated health were the strongest predictors of psychological well-being. Those that indicated that they had attended at least some college scored significantly higher across all dimensions of psychological well-being compared to those who had a high school degree or less, and higher self-rated health scores were associated with significantly higher psychological well-being scores. Sex and marital status were also robust predictors. Being female was associated with a lower score on autonomy (−0.863, *p* < 0.001) but a higher score on personal growth (0.928, *p* < 0.001), positive relations with others (2.130, *p* < 0.001), purpose in life (0.561, *p* < 0.001), and self-acceptance (0.212, *p* < 0.05). Being married was associated with higher scores on personal growth (0.378, *p* < 0.01), positive relations with others (1.237, *p* < 0.001), purpose in life (1.082, *p* < 0.001), and self-acceptance (0.707, *p* < 0.001). Increased age predicted lower scores on personal growth (−0.040, *p* < 0.05) and purpose in life (−0.075, *p* < 0.001), while increased household size predicted lower environmental mastery scores only (−0.238, *p* < 0.01). Being employed was significantly associated with higher scores on personal growth (0.449, *p* < 0.001), positive relations with others (0.362, *p* < 0.05), and purpose in life (0.597, *p* < 0.001).

The significant associations between the elder mistreatment variables and the outcomes did change with the inclusion of the demographic controls, but not substantially. Notable differences in the full models include physical abuse was no longer a significant predictor of environmental mastery, feeling exploited while buying became a significant predictor of autonomy (−0.514, *p* < 0.05), and feeling exploited while selling no longer significantly predicted purpose in life. Feeling someone had exerted excessive control over you in the past year and being worried you made a donation to an illegitimate charity both significantly predicted lower scores across all dimensions of psychological well-being even when accounting for the demographic controls.

The R^2^ values in the full models improved compared to the models without controls, suggesting their inclusion increased the models’ predictive power. The full models appeared to explain the most amount of variance in environmental mastery (15.4%) and purpose in life (12.6%).

## 4. Discussion

Elder mistreatment is a public health concern given its prevalence in the US and globally [2,4] as well as its measured impacts on physical and mental health [7,8,9,10,11,12,13,14,15,16,17]. This project contributes to the growing literature on elder mistreatment by examining the relationship between varied forms of mistreatment and eudaimonic measures of psychological well-being among older Americans. OLS regression analysis reveals that elder mistreatment is significantly associated with eudemonic dimensions of psychological well-being including autonomy, environmental mastery, personal growth, positive relations with others, purpose in life, and self-acceptance. These relationships were negative such that experiences of elder mistreatment were associated with significantly lower scores on psychological well-being with one exception—having belongings seized or withheld in the past year was associated with higher scores on autonomy. Most of these associations held even when demographic control variables were introduced into the models.

Individually, experiences with excessive control and exploitive solicitation (i.e., feeling taken advantage of by a potentially illegitimate charity) were the most consistent predictors of lower psychological well-being, as both forms of mistreatment had significant associations with all eudaimonic dimensions. As defined by Ryff [27,28,29,30,31], eudaimonic well-being emphasizes such constructs as individuation, development, and function. It thus makes sense that being the victim of excessive control would impact psychological well-being, as this type of mistreatment limits individuation, development, and function at its core. In addition, being taken advantage of via exploitive solicitation may motivate an older adult to re-evaluate their awareness and ability to differentiate between the legitimate and illegitimate, thus negatively impacting their sense of control and by extension their psychological well-being. While not as robust, feeling exploited while purchasing and selling did have negative associations with psychological well-being, supporting previous studies that examined the impacts of financial mistreatment among older adults [24].

The positive association between having belongings seized/withheld and autonomy is puzzling. Given that higher autonomy implies increased self-determination and confidence in living by one’s own convictions, the expectation is that any form of elder mistreatment would limit a person’s confidence and, by extension, their feelings of autonomy. The positive association with having belongings seized/withheld contradicts expectations and the other findings in this project. It is possible this is an anomaly, but further investigation is warranted.

While several theories postulate the motivations behind elder mistreatment and why older adults may be susceptible to mistreatment, a majority of these theories (e.g., caregiver stress theory, social exchange theory, political economic theory) emphasize a lack of control among victims and a reliance on others [25,26]. As an example, caregiver stress theory argues that, in caregiver/care recipient dyads, older adults that rely heavily on caregivers are susceptible to elder mistreatment from caregivers that are overwhelmed and stressed [26]. It is difficult to directly apply a single elder mistreatment theory to explain the results of this project, as most theories employ specific perspectives (e.g., caregiving perspectives) that fall out of the purview of this work. The findings of this project, however, did find that mistreatment in the form of excessive control was one of the more prevalent forms of mistreatment and had significant associations with all dimensions of psychological well-being. In this way, the findings of this study help illustrate what most elder mistreatment theories opine—that the negative consequences of elder mistreatment are largely the product of a loss of independence and control in one’s life.

The findings of this project have practical application both in risk assessment and in promotion of eudaimonia. The findings illustrate how varied forms of elder mistreatment are significantly associated not only with eudaimonia on the whole, but also with specific dimensions of eudaimonia. As an example, in the full models of this project there was a significant association between feelings of personal growth and two elder mistreatment predictors: excessive control and feeling exploited while making charitable donations. Thus, if a victim of excessive control or financial exploitation presents to stakeholders in the care of older adults, it may behoove these professionals to assess the potential impacts of this mistreatment on psychological well-being (e.g., personal growth). Similarly, if an older adult presents to a health professional with decreased feelings of personal growth, it is possible that this is a consequence of excessive control over their lives or financial exploitation. In this way, professional stakeholders in the care of older adults may be better equipped to identify the potential source of decreased eudaimonia. Professional stakeholders may also be better equipped to address these sources to promote better psychological well-being (e.g., if an older adult experiences lower psychological well-being as a result of excessive control, interventions can focus on increasing independence).

While this project makes a significant contribution to the literature on elder mistreatment, it is not without its limitations. Despite the data coming from a longitudinal study, only one wave contained the predictors of interest; as such, this is a cross-sectional project wherein causation can only be inferred and not proven (e.g., reverse-causation is possible in that lower psychological well-being can increase susceptibility to elder mistreatment). In addition, the sample is not representative of the entire US older adult population (e.g., there is an oversample of those with high school degrees given how the graduate sample was recruited, and while race/ethnicity was not controlled for, it is known that the sample is substantially White non-Hispanic), thus limiting generalizability. Given that respondents may have been more likely to provide complete data if their responses were more favorable, the process of listwise deletion to produce the analytic sample may have skewed the results in favor of those with fewer instances of elder mistreatment and/or those with better psychological well-being. This, too, may limit generalizability. Finally, the R^2^ values of the full regression models were relatively low, suggesting additional predictive variables can be included to explain more of the variance in psychological well-being scores.

Future studies should account for these limitations while also exploring potential mediating/moderating variables (e.g., social support). Future work should also examine whether psychological well-being mediates the relationship between elder mistreatment and physical/mental health outcomes. Given the literature on how psychological well-being impacts health [33,34,35,36,37,38,39], it is possible elder mistreatment may influence physical/mental health outcomes *through* changes in eudaimonic measures such as environmental mastery. Finally, future studies should examine how interventions designed to promote psychological well-being such as Lighten UP! [40,41] can be tailored and best applied to victims of elder mistreatment. Professionals who work with elder mistreatment victims (e.g., social workers, psychiatrists, etc.) may benefit from these types of intervention studies as results may elucidate strategies and practices that can better serve their clientele.

## 5. Conclusions

This project examined the associations between elder mistreatment and eudaimonic measures of psychological well-being among a sample of older Americans. Using data from the 2011 wave of the WLS, this project found that various forms of elder mistreatment significantly predicted psychological well-being, such that instances of elder mistreatment were generally associated with lower well-being. While the negative impacts of elder mistreatment on health are well-documented, this project addresses a gap in the literature in that it finds a yet unseen consequence of mistreatment. These findings suggest programs designed to help victims of elder mistreatment should account for potentially diminished eudaimonic well-being.

## Figures and Tables

**Table 1:** Measurement of psychological well-being.

Dimension	Items Per Dimension	Sample Item	Sum Ranges ^1^
*Autonomy*	5	“To what extent do you agree that you have confidence in your opinions even if they are contrary to the general consensus?”	0–25
*Environmental mastery*	5	“To what extent do you agree that you are quite good at managing the many responsibilities of your daily life?”	0–25
*Personal growth*	5	“To what extent do you agree that you have developed a lot as a person over time?”	0–25
*Positive relations with others*	6	“To what extent do you agree that you enjoy personal and mutual conversations with family members and friends?”	0–30
*Purpose in life*	6	“To what extent do you agree that you are an active person carrying out the plans you set for yourself?”	0–30
*Self-acceptance*	5	“To what extent do you agree that in general, you feel confident and positive about yourself?”	0–25

^1^ For all dimensions, higher scores indicate higher levels of psychological well-being.

**Table 2:** Measurement of elder mistreatment experiences.

Type of Mistreatment ^1^	Question Wording: “In the Past 12 Months …”
*Excessive control*	“…have you felt there is someone who is too controlling over your daily decisions and life?”
*Insults*	“…has anyone insulted you or put you down?”
*Seizures or withholds*	“…has anyone taken your money or belongings without your permission or prevented you from getting them even when you ask?”
*Physical abuse*	“…has anyone hit, kicked, slapped, or thrown things at you?”
*Blocked access*	“…has anyone intentionally prevented you from having things you need, such as medication, food, money, or personal care?”
*Exploited while buying*	“…have you made a purchase, either over the telephone or in person, where you later felt taken advantage of or ‘scammed’?”
*Exploited while selling*	“…have you sold a major possession, such as jewelry, where you later felt taken advantage of or ‘scammed’?”
*Exploitive solicitation*	“…have you made a donation to a charitable organization, either over the telephone, in person, or by mail where you later worried that the organization was not legitimate or ‘on the level’?”

^1^ All elder mistreatment items were scored such that 0 = *No*, 1 = *Yes*.

**Table 3:** Sample descriptives ^1^.

Predictor	Mean (SD) or %	Outcome	Mean (SD) or %
Age	71.38 (2.90)	Psychological well-being dimensions	
Female	53.59%	*Autonomy*	17.75 (3.67)
Some college	53.75%	*Environmental mastery*	19.70 (3.71)
Married	72.22%	*Personal growth*	19.50 (3.72)
Household members	1.90 (0.70)	*Positive relations with others*	23.06 (5.21)
Employed	27.01%	*Purpose in life*	21.87 (4.69)
Self-rated health	3.00 (0.67)	*Self-acceptance*	19.51 (3.74)
Mistreatment experiences			
*Excessive control*	7.49%		
*Insults*	14.90%		
*Seizures or withholds*	1.84%		
*Physical abuse*	0.52%		
*Blocking access*	0.24%		
*Exploited while buying*	3.59%		
*Exploited while selling*	0.62%		
*Exploitive solicitation*	2.13%		

^1^*N* = 6158.

**Table 4:** Pearson correlation coefficients ^1^.

Variable	1	2	3	4	5	6	7	8	9	10	11	12	13	14	15	16	17	18	19	20	21
1. Age	-																				
2. Female	0.005	-																			
3. Some college	**−0.100**	**−0.102**	-																		
4. Married	**−0.113**	**−0.202**	**0.030**	-																	
5. Household members	**−0.093**	**−0.108**	0.013	**0.494**	-																
6. Employed	**−0.123**	**−0.120**	**0.080**	0.011	0.023	-															
7. Self-rated health	**−0.096**	**0.030**	**0.165**	**0.048**	−0.024	**0.099**	-														
8. Excessive control	**−0.038**	**0.026**	**0.027**	**0.068**	**0.064**	−0.008	**−0.083**	-													
9. Insults	**−0.090**	**0.084**	**0.070**	0.007	**0.025**	0.017	−0.024	**0.351**	-												
10. Seizures or withholds	−0.004	−0.016	0.010	**−0.064**	**0.031**	0.001	**−0.034**	**0.127**	**0.120**	-											
11. Physical abuse	−0.021	−0.019	0.008	0.004	**0.075**	−0.008	−0.010	**0.143**	**0.103**	0.024	-										
12. Blocking access	−0.004	−0.020	0.019	**−0.028**	**0.026**	0.022	0.015	**0.099**	**0.063**	**0.116**	**0.042**	-									
13. Exploited while buying	−0.013	−0.011	**0.053**	**−0.028**	0.009	0.022	−0.011	**0.068**	**0.066**	**0.065**	0.010	**0.026**	-								
14. Exploited while selling	0.013	0.003	−0.010	−0.016	0.002	−0.006	−0.006	0.017	**0.043**	**0.036**	**0.052**	−0.004	**0.052**	-							
15. Exploitive solicitation	**0.034**	0.011	0.019	**−0.029**	0.004	−0.016	**−0.030**	**0.044**	**0.068**	**0.055**	0.005	0.016	**0.074**	0.017	-						
16. Autonomy	**−0.041**	**−0.130**	**0.153**	**0.038**	**0.034**	**0.032**	**0.135**	**−0.039**	**−0.026**	0.019	0.010	0.015	−0.024	−0.022	**−0.055**	-					
17. Environmental mastery	**−0.044**	0.005	**0.138**	−0.001	**−0.058**	**0.025**	**0.336**	**−0.174**	**−0.128**	**−0.051**	**−0.055**	**−0.034**	**−0.049**	**−0.046**	**−0.053**	**0.436**	-				
18. Personal growth	**−0.075**	**0.094**	**0.207**	**0.027**	−0.010	**0.077**	**0.246**	**−0.065**	−0.017	−0.012	−0.016	0.004	−0.007	**−0.026**	**−0.034**	**0.417**	**0.586**	-			
19. Positive relations with others	**−0.029**	**0.172**	**0.040**	**0.076**	**0.025**	**0.027**	**0.196**	**−0.097**	**−0.085**	**−0.043**	**−0.056**	−0.013	**−0.051**	**−0031**	**−0.043**	**0.313**	**0.533**	**0.549**	-		
20. Purpose in life	**−0.101**	0.020	**0.206**	**0.108**	**0.037**	**0.092**	**0.281**	**−0.069**	−0.018	−0.021	−0.002	−0.010	**−0.025**	−0.025	**−0.049**	**0.399**	**0.598**	**0.659**	**0.549**	-	
21. Self-acceptance	−0.012	0.000	**0.100**	**0.089**	**0.027**	**0.041**	**0.265**	**−0.112**	**−0.110**	**−0.037**	**−0.035**	−0.013	**−0.047**	**−0.042**	**−0.059**	**0.427**	**0.684**	**0.594**	**0.541**	**0.577**	-

^1^ Coefficients highlighted in **bold** indicate *p* < 0.05 (two-tailed).

**Table 5:** OLS regression models with elder mistreatment predicting psychological well-being ^1^.

Predictor	Autonomy	Environmental Mastery	Personal Growth	Positive Relations	Purpose in Life	Self-Acceptance
*Excessive control*	−0.552 **	−1.956 ***	−0.933 ***	−1.348 ***	−1.257 ***	−1.118 ***
*Insults*	−0.150	−0.715 ***	0.104	−0.723 ***	0.142	−0.776 ***
*Seizures or withholds*	0.742*	−0.506	−0.023	−0.881	−0.362	−0.230
*Physical abuse*	0.725	−1.386 *	−0.360	−2.892 **	0.379	−0.616
*Blocking access*	1.283	−0.878	0.797	0.300	−0.157	0.170
*Exploited while buying*	−0.329	−0.572 *	−0.025	−1.057 **	−0.399	−0.613 *
*Exploited while selling*	−0.928	−1.737 **	−1.032	−1.325	−1.555 *	−1.502 *
*Exploitive solicitation*	−1.299 ***	−0.984 **	−0.799 *	−1.152 *	−1.423 **	−1.177 ***
Adjusted R^2^	0.005	0.040	0.005	0.017	0.007	0.022

^1^ Significant relationships shown as: * *p* < 0.05, ** *p* < 0.01, *** *p* < 0.001.

**Table 6:** Full OLS regression models with demographic controls included ^1^.

Predictor	Autonomy	Environmental Mastery	Personal Growth	Positive Relations	Purpose in Life	Self-Acceptance
Age	−0.021	−0.027	−0.040 *	−0.006	−0.075 ***	0.027
Female	−0.863 ***	0.072	0.928 ***	2.130 ***	0.561 ***	0.212 *
Some college	0.916 ***	0.735 ***	1.362 ***	0.381 **	1.560 ***	0.532 ***
Married	−0.065	0.066	0.378 **	1.237 ***	1.082 ***	0.707 ***
Household members	0.136	−0.238 **	−0.093	0.047	−0.044	0.014
Employed	−0.050	−0.105	0.449 ***	0.362 *	0.597 ***	0.149
Self-rated health	0.623 ***	1.683 ***	1.089 ***	1.298 ***	1.611 ***	1.332 ***
Mistreatment experiences						
*Excessive control*	−0.397 *	−1.574 ***	−0.751 ***	−1.239 ***	−1.041 ***	−0.932 ***
*Insults*	−0.132	−0.828 ***	−0.194	−1.051 ***	−0.142	−0.836 ***
*Seizures or withholds*	0.742 *	−0.231	0.235	−0.263	0.222	0.003
*Physical abuse*	0.575	−1.156	−0.133	−2.410 **	0.659	−0.682
*Blocking access*	0.679	−1.485	0.485	0.476	−0.700	−0.149
*Exploited while buying*	−0.514 *	−0.628 **	−0.124	−0.956 **	−0.549	−0.615 *
*Exploited while selling*	−0.832	−1.611 **	−0.912	−1.340	−1.012	−1.512 **
*Exploitive solicitation*	−1.269 ***	−0.782 *	−0.679 *	−0.951 *	−1.138 **	−1.037 **
Adjusted R^2^	0.054	0.154	0.109	0.094	0.126	0.098

^1^ Significant relationships shown as: * *p* < 0.05, ** *p* < 0.01, *** *p* < 0.001.

## References

[1] Council on Scientific Affairs (1987). Elder abuse and neglect. JAMA.

[2] Lachs M.S., Pillemer K.A. (2015). Elder abuse. N. Engl. J. Med..

[3] Connolly M.T., Brandl B., Breckman R. (2014). The Elder Justice Roadmap: A Stakeholder Initiative to Respond to an Emerging Health, Justice, Financial and Social Crisis.

[4] Yon M.Y., Mikton C.R., Gassoumis Z.D., Wilber K.H. (2017). Elder abuse prevalence in community settings: A systematic review and meta-analysis. Lancet Glob. Health.

[5] Swagerty D.L., Takahashi P.Y., Evans J.M. (1999). Elder mistreatment. Am. Fam. Physician.

[6] Bond M., Butler K.H. (2013). Elder Abuse and Neglect. Clin. Geriatr. Med..

[7] Baker M.W. (2007). Elder Mistreatment: Risk, Vulnerability, and Early Mortality. J. Am. Psychiatr. Nurses Assoc..

[8] Baker M.W., Lacroix A.Z., Wu C., Cochrane B.B., Wallace R., Woods N.F. (2009). Mortality Risk Associated with Physical and Verbal Abuse in Women Aged 50 to 79. J. Am. Geriatr. Soc..

[9] Lachs M.S., Williams C.S., O’Brien S., Pillemer K.A., Charlson M.E. (1998). The Mortality of Elder Mistreatment. JAMA.

[10] Li M., Liang Y., Dong X. (2019). Different Definitions of Elder Mistreatment and Mortality: A Prospective Cohort Study From 2011 to 2017. J. Am. Geriatr. Soc..

[11] Schofield M.J., Powers J.R., Loxton D.J. (2013). Mortality and Disability Outcomes of Self-Reported Elder Abuse: A 12-Year Prospective Investigation. J. Am. Geriatr. Soc..

[12] Wong J.S., Waite L.J. (2017). Response to Acierno’s comments on Wong and Waite, “Elder mistreatment predicts later physical and psychological health: Results from a national longitudinal study”. J. Elder Abus. Negl..

[13] Acierno R., Dha M.A.H.-T., Anetzberger G.J., Loew D., Muzzy W. (2017). The National Elder Mistreatment Study: An 8-year longitudinal study of outcomes. J. Elder Abus. Negl..

[14] Acierno R., Watkins J., Hernandez-Tejada M.A., Muzzy W., Frook G., Steedley M., Anetzberger G. (2018). Mental Health Correlates of Financial Mistreatment in the National Elder Mistreatment Study Wave II. J. Aging Health.

[15] Chao Y.-Y., Li M., Lu S.-E., Dong X. (2020). Elder mistreatment and psychological distress among U.S. Chinese older adults. J. Elder Abus. Negl..

[16] Fulmer T., Rodgers R.F., Pelger A. (2014). Verbal Mistreatment of the Elderly. J. Elder Abus. Negl..

[17] Choi Y.-J., O’Donnell M.L., Choi H.-B., Jung H.-S., Cowlishaw S. (2018). Associations among Elder Abuse, Depression and PTSD in South Korean Older Adults. Int. J. Environ. Res. Public Health.

[18] Amstadter A.B., Zajac K., Strachan M., Hernandez M.A., Kilpatrick D.G., Acierno R. (2011). Prevalence and correlates of elder mistreatment in South Carolina: The South Carolina elder mistreatment study. J. Interpers. Violence.

[19] Pillemer K., Burnes D., Riffin C., Lachs M.S. (2016). Elder Abuse: Global Situation, Risk Factors, and Prevention Strategies. Gerontologist.

[20] Sathya T., Premkumar R. (2020). Association of functional limitations and disability with elder abuse in India: A cross-sectional study. BMC Geriatr..

[21] Badenes-Ribera L., Fabris M.A., Longobardi C. (2019). Elder Mistreatment in an Italian Population: Prevalence and Correlates. Int. J. Aging Hum. Dev..

[22] Dong X.Q., Chen R., Fulmer T.T., Simon M.A. (2014). Prevalence and Correlates of Elder Mistreatment in a Community-Dwelling Population of U.S. Chinese Older Adults. J. Aging Health.

[23] Piña-Escudero S.D., Chodos A., Weinstein C.A., Allen I.E., Ávila-Funes J.A., Ritchie C. (2020). Subjective cognitive decline and elder mistreatment in Mexican community-dwelling older adults. Arch. Gerontol. Geriatr..

[24] Acierno R., Hernandez M.A., Amstadter A.B., Resnick H.S., Steve K., Muzzy W., Kilpatrick D.G. (2010). Prevalence and Correlates of Emotional, Physical, Sexual, and Financial Abuse and Potential Neglect in the United States: The National Elder Mistreatment Study. Am. J. Public Health.

[25] Momtaz Y.A., Hamid T.A., Ibrahim R. (2013). Theories and measures of elder abuse. Psychogeriatrics.

[26] Mosqueda L., Burnight K., Gironda M.W., Moore A.A., Robinson J., Olsen B. (2016). The Abuse Intervention Model: A Pragmatic Approach to Intervention for Elder Mistreatment. J. Am. Geriatr. Soc..

[27] Ryff C.D. (1989). Happiness is everything, or is it? Explorations on the meaning of psychological well-being. J. Pers. Soc. Psychol..

[28] Ryff C.D. (2014). Psychological Well-Being Revisited: Advances in the Science and Practice of Eudaimonia. Psychother. Psychosom..

[29] Ryff C.D., Keyes C.L.M. (1995). The structure of psychological well-being revisited. J. Pers. Soc. Psychol..

[30] Ryff C.D., Singer B. (1996). Psychological Weil-Being: Meaning, Measurement, and Implications for Psychotherapy Research. Psychother. Psychosom..

[31] Ryan R.M., Deci E.L. (2001). On Happiness and Human Potentials: A Review of Research on Hedonic and Eudaimonic Well-Being. Annu. Rev. Psychol..

[32] Springer K.W., Pudrovska T., Hauser R.M. (2011). Does psychological well-being change with age? Longitudinal tests of age variations and further exploration of the multidimensionality of Ryff’s model of psychological well-being. Soc. Sci. Res..

[33] Boyle P.A., Barnes L.L., Buchman A.S., Bennett D.A. (2009). Purpose in Life Is Associated With Mortality among Community-Dwelling Older Persons. Psychosom. Med..

[34] Hill P.L., Turiano N.A. (2014). Purpose in Life as a Predictor of Mortality across Adulthood. Psychol. Sci..

[35] Boyle P.A., Buchman A.S., Barnes L.L., Bennett D.A. (2010). Effect of a Purpose in Life on Risk of Incident Alzheimer Disease and Mild Cognitive Impairment in Community-Dwelling Older Persons. Arch. Gen. Psychiatry.

[36] Kim E.S., Sun J.K., Park N., Peterson C. (2013). Purpose in life and reduced incidence of stroke in older adults: ‘The Health and Retirement Study’. J. Psychosom. Res..

[37] Kim E.S., Strecher V.J., Ryff C.D. (2014). Purpose in life and use of preventive health care services. Proc. Natl. Acad. Sci. USA.

[38] Friedman E.M., Ryff C.D. (2012). Living Well With Medical Comorbidities: A Biopsychosocial Perspective. J. Gerontol. Ser. B.

[39] Phelan C.H., Love G.D., Ryff C.D., Brown R.L., Heidrich S.M. (2010). Psychosocial predictors of changing sleep patterns in aging women: A multiple pathway approach. Psychol. Aging.

[40] Friedman E.M., Ruini C., Foy R., Jaros L., Sampson H., Ryff C.D. (2015). Lighten UP! A community-based group intervention to promote psychological well-being in older adults. Aging Ment. Health.

[41] Friedman E.M., Ruini C., Foy C.R., Jaros L., Love G., Ryff C.D. (2019). Lighten UP! A Community-Based Group Intervention to Promote Eudaimonic Well-Being in Older Adults: A Multi-Site Replication with 6 Month Follow-Up. Clin. Gerontol..

[42] Herd P., Carr D., Roan C. (2014). Cohort profile: Wisconsin longitudinal study (WLS). Int. J. Epidemiol..

[43] Hauser R.M., Sewell W.H., Herd P. (2019). Wisconsin Longitudinal Study (WLS) [Graduates, Siblings, and Spouses]: 1957–2019.

[44] Shrive F.M., Stuart H., Quan H., A Ghali W. (2006). Dealing with missing data in a multi-question depression scale: A comparison of imputation methods. BMC Med. Res. Methodol..

